# A stability-enhanced preconditioned Haar wavelet scheme for the solution of Poisson and Helmholtz equations

**DOI:** 10.1038/s41598-026-54376-5

**Published:** 2026-05-28

**Authors:** Avinash K., Harinakshi Karkera

**Affiliations:** Manipal Institute of Technology, Manipal Academy of Higher Education, Manipal, 576104 Karnataka India

**Keywords:** Helmholtz equation, Poisson equation, Condition number, Preconditioner, Haar wavelet, Krylov subspace method, Stability analysis, Engineering, Mathematics and computing, Physics

## Abstract

This study presents a modified numerical framework based on scale-3 Haar wavelets for the solution of elliptic partial differential equations, namely the Poisson and the Helmholtz equations. The spatial derivatives are discretized using Haar wavelet expansions and extended to two-dimensions through the Kronecker tensor product, with boundary conditions enforced via integration constants. A theoretical convergence analysis is conducted, and complemented by a rigorous stability investigation based on both classical and effective condition numbers to address ill-conditioning in the resulting linear systems at higher resolution levels. For the Poisson equation, the effectiveness of wavelet-based and multigrid preconditioners is examined, which shows that the multigrid preconditioner yields superior performance compared to the Haar wavelet preconditioner for scale-2 Haar discretization, while the Haar wavelet preconditioner performs better for scale-3 discretization when combined with the conjugate gradient method. For the Helmholtz equation, a shifted Laplacian preconditioner coupled with the GMRES solver is employed. Numerical simulations and spectral analyses, implemented in MATLAB R2025a, confirm the enhanced stability and convergence of the proposed preconditioned wavelet framework.

## Introduction

Time-harmonic wave propagation phenomena arises in numerous scientific and engineering disciplines, such as acoustics, electromagnetism, and structural dynamics, and their accurate numerical simulation is of considerable practical importance. When temporal dependence is eliminated through a harmonic ansatz and separation of variables, the governing wave equation reduces to a time-independent mathematical form that describes the spatial behavior of oscillatory fields. This resulting model is characterized by strong oscillations, which makes its numerical treatment substantially challenging, whence motivated extensive research on efficient and robust solution strategies. As the scalar counterpart of the time-harmonic Maxwell equations, this formulation serves as a cornerstone for modeling acoustic and electromagnetic wave propagation, and is mathematically represented by the Helmholtz equation,

1 $$\begin{aligned} -\Delta u-K^2u=f, \end{aligned}$$

where $K\in \mathbb {R}^{+}$ denotes the wave number, *u* is the unknown field variable, and *f* represents a prescribed source term, together with appropriate boundary conditions. In this study, particular attention is given to the challenging regime in which $K^2$ is close to an eigenvalue of the Laplacian operator — a situation that often leads to severe numerical difficulties. Closely related to the Helmholtz equation is the Poisson equation,

2 $$\begin{aligned} -\Delta u=f, \end{aligned}$$

which arises as a limiting case when the wave number term vanishes and serves as a fundamental model in electrostatics, heat conduction, and incompressible fluid flow. The Poisson problem is typically better conditioned than the Helmholtz problem and is frequently used as a benchmark for assessing the stability and efficiency of numerical discretization and solution techniques. Many solvers and preconditioners developed for the Helmholtz equation are also motivated by, or compared against, their performance on the Poisson equation.

In the two-dimensional Helmholtz equation, the wave number *K* plays a decisive role in determining both the qualitative behavior of the solution and the associated numerical difficulty. For low wave numbers, corresponding to long wavelengths, the solution is smooth, and the operator behaves similarly to a Poisson-type problem, resulting in well-conditioned linear systems that can be efficiently solved using standard discretization and iterative techniques. As *K* increases, the solution becomes increasingly oscillatory, and the Helmholtz operator turns strongly indefinite, leading to rapidly growing condition numbers, eigenvalue clustering near the origin, and the onset of the pollution effect, which necessitates increasingly finer discretizations to maintain accuracy. Although the Helmholtz equation is mathematically well-defined for arbitrarily large *K*, in practice the attainable wave number is limited by computational cost, as the degrees of freedom in two-dimensions scales quadratically with *K*. Physically, small values of *K* correspond to low-frequency wave propagation characterized by smooth spatial variations and gradual energy transport. As *K* increases, the waves exhibit progressively shorter wavelengths, leading to pronounced interference and resonance effects that dominate the solution behavior. In this high-frequency regime, the governing model approaches the geometrical optics limit, thereby necessitating specialized numerical solvers and effective preconditioning strategies to ensure stable and efficient computation.

Upon discretization using any of the numerical methods such as finite difference method (FDM)^1,2^, finite element method (FEM)^3^, wavelet-based discretisation methods^4–10^ etc., both the Helmholtz and Poisson equations lead to large sparse linear systems of the form *Au=b*, where $A\in \mathbb {C}^{n\times n}$ is a sparse, non-singular matrix that may be symmetric in the case of Poisson and non-symmetric or non-Hermitian in the Helmholtz case. Here, $u\in \mathbb {C}^n$ is the unknown solution vector, and $b\in \mathbb {C}^n$ is the known vector. To achieve acceptable solution accuracy, especially for the Helmholtz equation, the number of degrees of freedom per wavelength must be sufficiently large and must increase faster than the wave number in order to control the pollution effect^11^. As a result, high-frequency Helmholtz problems give rise to extremely large and often ill-conditioned linear systems, posing significant challenges for efficient and robust numerical solution methods.

On the other hand, Haar wavelets consist of pairs of piecewise constant functions and represent the mathematically simplest form of orthonormal wavelets with compact support. Owing to their analytical tractability, Haar wavelet method has proven to be an effective tool for solving differential and integral equations. Chen and Hsiao’s^12^ seminal work on Haar wavelet analysis in dynamical systems introduced the Haar operational matrix for the integration of Haar vector functions. Extending this framework, Hsiao^13^ subsequently developed the Haar product matrix and the coefficient matrix, which facilitated the state analysis of linear time-delayed systems. Building on the pioneering work reported in^14^, Lepik^15^ introduced the use of two-dimensional Haar wavelets for obtaining numerical solutions of partial differential equations. In addition, Shi et al.^9,16^ developed robust Haar wavelet based computational frameworks for the numerical solution of Poisson and biharmonic equations defined over two- and three-dimensional domains. An advanced extension of the classical Haar basis, known as scale-3 Haar wavelets, was later introduced by Mittal and Pandit^17–20^, incorporating symmetric and antisymmetric features that enhance approximation capability. In recent years, scale-3 Haar wavelets have garnered considerable attention from both the mathematical and engineering communities due to their proven effectiveness in addressing a wide spectrum of functional equations, including ODEs and PDEs.

Conditioning analysis plays a crucial and challenging role in the study of stability for sparse linear systems arising from numerical discretization methods^21^. A large condition number of the system matrix *A* indicates heightened sensitivity to perturbations and numerical errors, rendering the system ill-conditioned. In such situations, the effective condition number is often employed to provide a more informative measure of numerical behavior, as suggested by Li et al.^22–24^. To mitigate the adverse effects of ill-conditioning, preconditioning techniques are introduced, after which the resulting preconditioned systems are efficiently solved using Krylov subspace iterative methods. In the absence of preconditioning, the matrices arising from the discretization of elliptic (in perticular, Helmoltz equation) and real-world problems may exhibit unfavourable eigenvalue distributions. For the Helmoltz equation, especially at higher wave numbers, the system becomes indefinite and highly oscillatory, leading to slow convergence or stagnation of Krylov subspace iterative methods. In such cases, the number of iterations increses significantly, and in some instances, convergence cannot be achieved or may diverge away from the solution. The introduction of preconditioning improves the clustering of eigenvalues near unity and reduces the condition number of the system, thereby accelerating convergence and enhancing numerical stability. This becomes perticularly important in real-world applications such as acoustic wave propagation, electromagnetic scattering, and seismic imaging, where large scale Helmoltz problems are routinely encountered. A variety of effective preconditioners have been proposed in the literature, including wavelet-based preconditioners employing complete and incomplete discrete wavelet transforms^25^, multigrid and algebraic multigrid (AMG) approaches^26,27^, shifted Laplacian preconditioners with damping^28^, and many other preconditioners^29,30^.

The conjugate gradient (CG) method, proposed by Hestenes and Stiefel^31^, was the earliest Krylov subspace technique developed for the solution of linear systems. Under exact arithmetic, CG is guaranteed to converge in at most *n* iterations. Consequently, although CG was originally introduced together with the closely related Lanczos method^32^, as an iterative algorithm, it was for many years regarded by mathematicians as a direct solver rather than an iterative one, in contrast to the perspective commonly held by engineers and physicists. In practice, CG often proved less competitive than other available solution strategies in this role. A turning point came with John Reid’s observation^33^ that highly accurate approximations to the solution can be achieved in far fewer than *n* iterations. This realization revived interest in Krylov subspace methods as genuinely iterative techniques and stimulated the development and systematic analysis of a wide range of algorithms, including the Bi-Conjugate Gradient (BiCG), Conjugate Gradient Squared (CGS), Bi-Conjugate Gradient Stabilized (BiCGSTAB), and the restarted Generalized Minimal Residual (GMRES) method^34^, which are now widely employed for solving large-scale linear systems.

The literature review bears witness to the fact that there is a lack of detailed studies on extracting the qualitative information about conditioning analysis of the resulting system via the Haar wavelet method, which are essential for numerical stability. To address the limitations of existing wavelet-based approaches, this work finds a balance shift to the qualitative information about the behavior of solutions, convergence, stability, efficiency in tackling PDEs, etc., rather than just finding a numerical solution or quantitative information about it. Hence, our primary contribution in this work is threefold. Firstly, a modified scale-3 Haar wavelet framework with convergence analysis. Secondly, we exemplify the classical and effective condition number analysis and concrete implementation process of a suitable preconditioner with Krylov subspace solver. Lastly, we draw some potential observations of the analysis that provide a deeper understanding of the stability.

The paper is structured into six sections. Following the introduction, Section “Haar wavelets” presents the preliminaries of Haar wavelets, while Section "Haar wavelet method" unfolds the modified wavelet method. Sections "Convergence of scale-3 Haar wavelet method" and “Conditioning analysis” focus on the analysis of system conditioning, preconditioning strategies, and Krylov subspace solvers, along with numerical results for three partial differential equations. Finally, Section “Numerical experiments” concludes the paper with a summary of the main findings and observations.

## Haar wavelets

### Scale-2 Haar wavelets

In wavelet analysis, two key functions namely the scaling function and the wavelet function play a vital role. These functions generate a family of successive wavelet functions that decompose any square-integrable functions. Sometimes, the scaling function is designated as the father wavelet, while the wavelet function is called the mother wavelet^35^. Consider the interval [*A*, *B*], and a maximum resolution level *J*. Define $M=2^J$ and the sub-interval length $\Delta t=\frac{B-A}{2M}$. The dilation parameter *j* ranging from 0 to *J* determines the level of compression, while the translation parameter *k* ranging from 0 to *m-1* shifts the wavelet’s position within the interval. Here $m=2^j$ and *i=m+k+1* is the wavelet number. Haar scaling function for *i=1* is given by:

3 $$\begin{aligned} h_1(t)={\left\{ \begin{array}{ll} 1 & \text {for } t\in \left[ A,B\right) \\ 0 & \text {otherwise}.\end{array}\right. } \end{aligned}$$

Further, when *m=1* and *k=0*, the index *i=2* represents the Haar mother wavelet. The subsequent wavelets can be identified by:

4 $$\begin{aligned} h_i(t)={\left\{ \begin{array}{ll} 1 & \text {for } t\in \left[ \varsigma _1(i),\varsigma _2(i) \right) \\ -1 & \text {for } t\in \left[ \varsigma _2(i),\varsigma _3(i) \right) \\ 0 & \text {otherwise},\end{array}\right. } \end{aligned}$$

where $\varsigma _1(i)=A+2k\mu \Delta t,\hspace{0.1cm}\varsigma _2(i)=A+(2k+1)\mu \Delta t,\hspace{0.1cm}\varsigma _3(i)=A+2(k+1)\mu \Delta t$ and $\mu =\frac{M}{m}$. The $r^{th}$ order integral of Haar functions are defined as follows:

5 $$\begin{aligned} p_{r,i}(t)=\int _A^t\int _A^t\cdots \int _A^th_i(z)dz^r=\frac{1}{(r-1)!}\int _{A}^{t}(t-z)^{r-1}h_i(z)dz. \end{aligned}$$

The explicit form of $p_{r,i}(t)$ for *i=2,3,… ,2M* is given by

6 $$\begin{aligned} p_{r,i}(t)={\left\{ \begin{array}{ll} 0 & \text {for } t < \varsigma _1(i) \\ \frac{1}{r!}(t-\varsigma _1(i))^r & \text {for } t\in \left[ \varsigma _1(i),\varsigma _2(i) \right) \\ \frac{1}{r!}\left\{ (t-\varsigma _1(i))^r-2(t-\varsigma _2(i))^r\right\} & \text {for } t\in \left[ \varsigma _2(i),\varsigma _3(i) \right) \\ \frac{1}{r!}\left\{ (t-\varsigma _1(i))^r-2(t-\varsigma _2(i))^r+(t- \varsigma _3(i))^r\right\} & \text {for } t> \varsigma _3(i). \end{array}\right. } \end{aligned}$$

For the case *i=1* with $\varsigma _1=A$, $\varsigma _2=\varsigma _3=B:$

7 $$\begin{aligned} p_{r,1}(t)= \frac{1}{r!}(t-A)^r. \end{aligned}$$

### Scale-3 Haar wavelets

For any *z∈ [A,B]* with maximal resolution level *J*, define $M=3^J$ and sub-interval length as $\Delta z=\frac{B-A}{3M}$. Let *j=0,1,2,… ,J* be the dilation parameter, *k=0,1,2,… ,m-1* be the translation parameter, where $m=3^j$ and index *i* gives the relation between *m* and *k*. The initial index value, *i=1* is assigned for scaling function defined as

8 $$\begin{aligned} h_1(z)={\left\{ \begin{array}{ll} 1 & \text {for } z\in \left[ A,B\right) \\ 0 & \text {otherwise}.\end{array}\right. } \end{aligned}$$

For *i>1* the even indices (*i = m + 2k + 1*) correspond to symmetric wavelets denoted by $\psi ^{(1)}(z)$, while the odd indices (*i = m + 2k + 2*) represent antisymmetric wavelets denoted by $\psi ^{(2)}(z)$. Both are defined in function form as:

9 $$\begin{aligned} \psi _i^{(1)}(z)=\frac{1}{\sqrt{2}}{\left\{ \begin{array}{ll} -1 & \text {for } z\in \left[ \boldsymbol{\rho }_1(i),\boldsymbol{\rho }_2(i) \right) \\ 2 & \text {for } z\in \left[ \boldsymbol{\rho }_2(i),\boldsymbol{\rho }_3(i) \right) \\ -1 & \text {for } z\in \left[ \boldsymbol{\rho }_3(i),\boldsymbol{\rho }_4(i) \right) ,\end{array}\right. } \end{aligned}$$

10 $$\begin{aligned} \psi _i^{(2)}(z)=\sqrt{\frac{3}{2}}{\left\{ \begin{array}{ll} 1 & \text {for } z\in \left[ \boldsymbol{\rho }_1(i),\boldsymbol{\rho }_2(i) \right) \\ 0 & \text {for } z\in \left[ \boldsymbol{\rho }_2(i),\boldsymbol{\rho }_3(i) \right) \\ -1 & \text {for } z\in \left[ \boldsymbol{\rho }_3(i),\boldsymbol{\rho }_4(i) \right) ,\end{array}\right. } \end{aligned}$$

where

$$\begin{aligned} \boldsymbol{\rho }_1(i)&=A+(B-A)\frac{k}{m};\qquad \qquad \qquad \quad \quad \boldsymbol{\rho }_3(i)=A+(B-A)\frac{3k+2}{3m};\\ \boldsymbol{\rho }_2(i)&=A+(B-A)\frac{3k+1}{3m};\qquad \qquad \qquad \boldsymbol{\rho }_4(i)=A+(B-A)\frac{k+1}{m}. \end{aligned}$$

The $r^{th}$ order integrals of scale-3 Haar function are given by

11 $$\begin{aligned} p_{r,1}(z)={\left\{ \begin{array}{ll} \frac{(z-A)^r}{r!}& \text {for } z\in \left[ A,B \right) \\ 0 & \text {otherwise,}\end{array}\right. }\end{aligned}$$

12 $$\begin{aligned} \psi _{r,i}^{(1)}(z)=\frac{1}{\sqrt{2}}{\left\{ \begin{array}{ll} \frac{-(z-\boldsymbol{\rho }_1(i))^r}{r!} & \text {for } z\in \left[ \boldsymbol{\rho }_1(i),\boldsymbol{\rho }_2(i) \right) \\ \frac{-(z-\boldsymbol{\rho }_1(i))^r+3(z-\boldsymbol{\rho }_2(i))^r}{r!} & \text {for } z\in \left[ \boldsymbol{\rho }_2(i),\boldsymbol{\rho }_3(i) \right) \\ \frac{-(z-\boldsymbol{\rho }_1(i))^r+3(z-\boldsymbol{\rho }_2(i))^r-3(z-\boldsymbol{\rho }_3(i))^r}{r!} & \text {for } z\in \left[ \boldsymbol{\rho }_3(i),\boldsymbol{\rho }_4(i) \right) \\ \frac{-(z-\boldsymbol{\rho }_1(i))^r+3(z-\boldsymbol{\rho }_2(i))^r-3(z-\boldsymbol{\rho }_3(i))^r+(z-\boldsymbol{\rho }_4(i))^r}{r!} & \text {for } z\in \left[ \boldsymbol{\rho }_4(i),B \right) , \end{array}\right. } \end{aligned}$$

13 $$\begin{aligned} \psi _{r,i}^{(2)}(z)=\sqrt{\frac{3}{2}}{\left\{ \begin{array}{ll} \frac{(z-\boldsymbol{\rho }_1(i))^r}{r!} & \text {for } z\in \left[ \boldsymbol{\rho }_1(i),\boldsymbol{\rho }_2(i) \right) \\ \frac{(z-\boldsymbol{\rho }_1(i))^r-(z-\boldsymbol{\rho }_2(i))^r}{r!} & \text {for } z\in \left[ \boldsymbol{\rho }_2(i),\boldsymbol{\rho }_3(i) \right) \\ \frac{(z-\boldsymbol{\rho }_1(i))^r-(z-\boldsymbol{\rho }_2(i))^r-(z-\boldsymbol{\rho }_3(i))^r}{r!} & \text {for } z\in \left[ \boldsymbol{\rho }_3(i),\boldsymbol{\rho }_4(i) \right) \\ \frac{(z-\boldsymbol{\rho }_1(i))^r-(z-\boldsymbol{\rho }_2(i))^r-(z-\boldsymbol{\rho }_3(i))^r+(z-\boldsymbol{\rho }_4(i))^r}{r!} & \text {for } z\in \left[ \boldsymbol{\rho }_4(i),B \right) . \end{array}\right. } \end{aligned}$$

## Haar wavelet method

Consider two elliptic PDEs of the form

14 $$\begin{aligned} \frac{\partial ^2 u(x,y)}{\partial x^2}+\frac{\partial ^2 u(x,y)}{\partial y^2}=g(x,y) \hspace{0.1cm};\hspace{0.3cm} (x,y)\in \Omega \end{aligned}$$

15 $$\begin{aligned} \frac{\partial ^2 u(x,y)}{\partial x^2}+\frac{\partial ^2 u(x,y)}{\partial y^2}+K^2u(x,y)=\tilde{g}(x,y) \hspace{0.1cm};\hspace{0.3cm} (x,y)\in \Omega \end{aligned}$$

with prescribed initial and boundary conditions. Equation (14) represents a Poisson equation whereas (15) represents a Helmholtz problem. The function *u*(*x*, *y*) and its derivatives are approximated using scale-3 HW. First, the *y* variable is kept fixed, and approximation of the highest ordered partial derivative with respect to *x* is carried out through the grid line parallel to *x*-axis, as follows:

16 $$\begin{aligned} \frac{\partial ^2 u(x)}{\partial x^2}= & c_1h_1+\sum _{even\hspace{0.1cm} index-i}^{3M} c_i\psi _i^{(1)}(x)+\sum _{odd\hspace{0.1cm} index-i}^{3M} c_i\psi _i^{(2)}(x),\end{aligned}$$

17 $$\begin{aligned} u(x)= & \sum _{i=1}^{3M}c_ip_{2,i}(x)+\frac{\partial u(A)}{\partial x} (x-A)+u(A), \end{aligned}$$

where $h_1(x)$, $\psi _i^{(1)}(x)$, $\psi _i^{(2)}(x)$, $p_{1,i}(x)$ and $p_{2,i}(x)$ constitute the Haar matrix and Haar integral matrices. At the collocation points $x_l$ ($1\le l\le 3M$)

18 $$\begin{aligned} u(x_l)= \begin{bmatrix} p_{2,1}(x_1) & p_{2,2}(x_1) & \cdots & p_{2,3M}(x_1) & x_1-A & 1\\ p_{2,1}(x_2) & p_{2,2}(x_2) & \cdots & p_{2,3M}(x_2) & x_2-A & 1\\ \vdots & \vdots & \ddots & \vdots & \vdots & \vdots \\ p_{2,1}(x_{3M}) & p_{2,2}(x_{3M}) & \cdots & p_{2,3M}(x_{3M}) & x_{3M}-A & 1\end{bmatrix}\begin{bmatrix}c\\ b_1\end{bmatrix}=Q_1\begin{bmatrix}c\\ b_1\end{bmatrix}, \end{aligned}$$

where $$c^T=(c_1,c_2,\ldots ,c_{3M})$$ and $$b_1^T=\big (\frac{\partial u(A)}{\partial x},u(A)\big )$$.

19 $$\begin{aligned} f_{ex1}= \begin{bmatrix} u(A)\\ u(B) \end{bmatrix}=\begin{bmatrix} p_{2,1}(A) & p_{2,2}(A) & \cdots & p_{2,3M}(A) & 0 & 1\\ p_{2,1}(B) & p_{2,2}(B) & \cdots & p_{2,3M}(B) & B-A & 1\\ \end{bmatrix}\begin{bmatrix}c\\ b_1\end{bmatrix}=Q_2\begin{bmatrix}a\\ b_1\end{bmatrix}, \end{aligned}$$

20 $$\begin{aligned} f_{\overline{ex1}}= \begin{bmatrix} u'(A)\\ u'(B) \end{bmatrix}=\begin{bmatrix} p_{1,1}(A) & p_{1,2}(A) & \cdots & p_{1,3M}(A) & 1 & 0\\ p_{1,1}(B) & p_{1,2}(B) & \cdots & p_{1,3M}(B) & 1 & 0\\ \end{bmatrix}\begin{bmatrix}c\\ b_1\end{bmatrix}=\overline{Q_2}\begin{bmatrix}a\\ b_1\end{bmatrix}. \end{aligned}$$

Equations (19) and (20) corresponds to the Dirichlet and Neumann boundary condition respectively. Combining (18) and boundary condition equations and substituting for $$\begin{bmatrix} c\\ b_1 \end{bmatrix}$$ in (16), reduces to

21 $$\begin{aligned} \begin{bmatrix} \frac{\partial ^2 u(x_1)}{\partial x^2}&\frac{\partial ^2 u(x_2)}{\partial x^2}&\cdots&\frac{\partial ^2 u(x_{3M})}{\partial x^2} \end{bmatrix}^T=\begin{bmatrix} H^T&0&0\end{bmatrix}Z^{-1}\begin{bmatrix}u\\ f_{ex1}\end{bmatrix}, \end{aligned}$$

22 $$\begin{aligned} \frac{\partial ^2 u}{\partial x^2}=\hat{H}Z^{-1}\begin{bmatrix}u\\ f_{ex1}\end{bmatrix}=B_1u+B_2f_{ex1}, \end{aligned}$$

where $$Z=\begin{bmatrix} Q_1 \\ Q_2 \end{bmatrix}$$. First *M* columns and the last two columns of the matrix $\hat{H}Z^{-1}$ are represented as $B_1$ and $B_2$ respectively. The approximation of $\frac{\partial ^2 u(x_l)}{\partial x^2}$ can be extended from 1D to 2D domain by making use of Kronecker tensor product as follows:

23 $$\begin{aligned} \mathbf {\frac{\partial ^2 u(x_l,y_k)}{\partial x^2}}=(B_1\otimes I_y)\mathbf {u(x_l,y_k)} +(B_2\otimes I_y)\mathbf {f_1}=\mathbf {R_x u(x_l,y_k)}+ \mathbf {k_x}, \end{aligned}$$

**Fig. 1:**
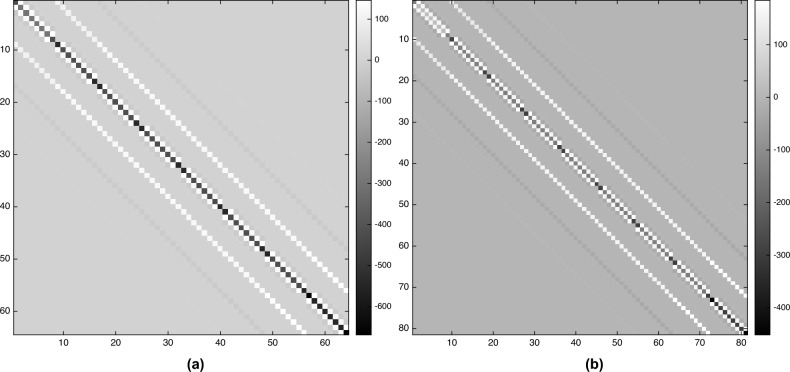
Representation of $(\mathbf {R_x}+\mathbf {R_y}+K^2\textbf{I})\textbf{u}$ obtained using the scale-2 Haar wavelet method with *J=2* (**a**) and the scale-3 Haar wavelet method with *J=1* (**b**).

where $\mathbf {f_1}$ is *6M× 1* column vector, $I_y$ is the identity matrix of order *3M× 3M*,

$$\begin{aligned} \textbf{u}^T=(u(x_1,y_1),\ldots ,u(x_1,y_{3M}),u(x_2,y_1),\ldots ,u(x_2,y_{3M}),\ldots ,u(x_{3M},y_1),\ldots ,u(x_{3M},y_{3M})), \end{aligned}$$

$$\begin{aligned} \Big (\mathbf {\frac{\partial ^2 u}{\partial x^2}}\Big )^T=\Big (\frac{\partial ^2 u(x_1,y_1)}{\partial x^2},\ldots ,\frac{\partial ^2 u(x_1,y_{3M})}{\partial x^2},\frac{\partial ^2 u(x_2,y_1)}{\partial x^2},\ldots ,\frac{\partial ^2 u(x_2,y_{3M})}{\partial x^2},\ldots ,\frac{\partial ^2 u(x_{3M},y_1)}{\partial x^2},\ldots ,\frac{\partial ^2 u(x_{3M},y_{3M})}{\partial x^2}\Big ). \end{aligned}$$

A similar procedure is carried out by fixing the variable *x* and approximating along the *y*-axis to get the final equation as

24 $$\begin{aligned} \mathbf {\frac{\partial ^2 u(x_l,y_k)}{\partial y^2}}=(I_x\otimes B_3)\mathbf {u(x_l,y_k)} +(I_x\otimes B_4)\mathbf {f_2}=\mathbf {R_y u(x_l,y_k)}+ \mathbf {k_y}, \end{aligned}$$

where $\mathbf {f_2}$ is *6M× 1* column vector, $I_x$ is the identity matrix of order *3M× 3M*.

On adding (23) and (24) along with $K^2\textbf{u}$ at 2D collocation points gives the following form for the Helmholtz problem:

25 $$\begin{aligned} (\mathbf {R_x}+\mathbf {R_y}+K^2\textbf{I})\textbf{u}=\mathbf {g(x_l,y_k)}-\mathbf {k_x}-\mathbf {k_y}, \end{aligned}$$

where $$\textbf{I}=I\otimes I$$.

The imagesc plots in Fig. 1 visualize the magnitude distribution of the operator $(\mathbf {R_x}+\mathbf {R_y}+K^2\textbf{I})\textbf{u}$ for scale-2 and scale-3 Haar wavelet discretizations. The observed block patterns reflect the underlying wavelet scales and resolution levels. This provides insight into the conditioning of the discrete operator and thereby helps in explaining the spectral properties and convergence behavior observed after preconditioning.

## Convergence of scale-3 Haar wavelet method

This section analyzes the convergence characteristics of the scale-3 Haar wavelet approximation associated with the proposed method, culminating in the result presented below:

### Theorem 1

*Let* *u*(*x*, *y*) *and* $u_{3M}(x,y)\in L^2([0,1]\times [0,1])$ *represent the exact and scale-3 Haar wavelet approximate solutions, respectively, and assume that they satisfy the Lipschitz condition. Then, the error estimate at the* $J^{th}$ *resolution level is given by*,

26 $$\begin{aligned} \parallel E_{3M}\parallel _2=\parallel u(x,y)-u_{3M}(x,y)\parallel _2\le \frac{KN}{(9\sqrt{18})3^{J}}. \end{aligned}$$

### Proof

Assume that $u_{3M}(x,y)$ is the approximate Haar solution using scale-3 HW and *u*(*x*, *y*) is the exact solution, then the error norm satisfies,

27 $$\begin{aligned} \parallel E_{3M}\parallel _2&=\parallel u(x,y)-u_{3M}(x,y) \parallel _2=\parallel u_{3M+1}(x,y)\parallel _2 \end{aligned}$$

28 $$\begin{aligned}&=\sum _{i,p=3M+1}^{\infty }a_{i,p}h_i(x)h_p(y). \end{aligned}$$

To find the coefficients $a_{i,p}$, following procedure is used:

29 $$\begin{aligned} a_{i,p}&=\int _{0}^{1}\int _{0}^{1}u(x,y)h_i(x)h_p(y)dxdy \end{aligned}$$

30 $$\begin{aligned}&=\langle h_i(x),\langle u(x,y),h_p(y)\rangle \rangle . \end{aligned}$$

Using orthogonality of Haar basis functions, square of error norm becomes

31 $$\begin{aligned} \parallel E_{3M}\parallel _2^2&=\int _{0}^{1}\int _{0}^{1}\left( \sum _{i,p=3M+1}^{\infty }a_{i,p}h_i(x)h_p(y)\right) ^2dxdy \end{aligned}$$

32 $$\begin{aligned}&=\sum _{i,p=3M+1}^{\infty }\sum _{s,q=3M+1}^{\infty }a_{i,p}a_{s,q}\left( \int _{0}^{1}h_i(x)h_s(x)dx \right) \left( \int _{0}^{1}h_p(y)h_q(y)dy\right) . \end{aligned}$$

Suppose that

33 $$\begin{aligned} \int _{0}^{1}h_i(x)h_s(x)dx= \int _{0}^{1}h_p(y)h_q(y)dy=K. \end{aligned}$$

This implies that

34 $$\begin{aligned} \parallel E_{3M}\parallel _2^2=K^2\sum _{i,p=3M+1}^{\infty }a_{i,p}^2. \end{aligned}$$

Choosing

35 $$\begin{aligned} W&=\langle u(x,y),h_p(y)\rangle \end{aligned}$$

36 $$\begin{aligned}&=3^{\frac{j}{2}}\int _{0}^{1}u(x,y)\phi (3^jy-k)dy, \end{aligned}$$

where $$\phi (3^jy-k)=\psi _i^{(1)}(y)+\psi _i^{(2)}(y)$$.

$$\begin{aligned} \therefore \hspace{0.2cm} W=3^{\frac{j}{2}} \left( \int _{\frac{k}{m}}^{\frac{k+\frac{1}{3}}{m}}\frac{-1}{\sqrt{2}}u(x,y)dy+\int _{\frac{k+\frac{1}{3}}{m}}^{\frac{k+\frac{2}{3}}{m}}\sqrt{2}u(x,y)dy+ \int _{\frac{k+\frac{2}{3}}{m}}^{\frac{k+1}{m}}\frac{-1}{\sqrt{2}}u(x,y)dy\right) \\ +3^{\frac{j}{2}}\left( \int _{\frac{k}{m}}^{\frac{k+\frac{1}{3}}{m}}\sqrt{\frac{3}{2}}u(x,y)dy-\int _{\frac{k+\frac{2}{3}}{m}}^{\frac{k+1}{m}} \sqrt{\frac{3}{2}}u(x,y)\right) . \end{aligned}$$

By using mean value theorem for integrals, *∃* $\beta _1\in \left( \frac{k}{m},\frac{k+\frac{1}{3}}{m}\right)$, $\beta _2\in \left( \frac{k+\frac{1}{3}}{m},\frac{k+\frac{2}{3}}{m}\right)$ and $\beta _3\in \left( \frac{k+\frac{2}{3}}{m}, \frac{k+1}{m} \right)$ such that,

37 $$\begin{aligned} W&=3^{\frac{j}{2}}\left[ \frac{1}{3m}\left( \frac{\sqrt{3}-1}{\sqrt{2}} \right) u(x,\beta _1) +\frac{\sqrt{2}}{3m}u(x,\beta _2)-\frac{1}{3m}\left( \frac{\sqrt{3}+1}{\sqrt{2}}u(x,\beta _3) \right) \right] \end{aligned}$$

38 $$\begin{aligned}&=\frac{1}{3^{\frac{j}{2}+1}}\left[ \sqrt{\frac{3}{2}}\left( u(x,\beta _1)- u(x,\beta _3)\right) -\frac{1}{\sqrt{2}}(u(x,\beta _1)-u(x,\beta _2))+\frac{1}{\sqrt{2}}(u(x,\beta _2)-u(x,\beta _3)) \right] . \end{aligned}$$

Consequently,

39 $$\begin{aligned} a_{i,p}&=\langle h_i(x),\langle u(x,y),h_p(y)\rangle \rangle \nonumber \\&=\langle h_i(x),W\rangle . \end{aligned}$$

Again using the definition of inner product and mean value theorem for integrals on (39), which yeilds

$$\begin{aligned} a_{i,p}&=\langle h_i(x),W\rangle \\&=3^{-j-2}\Big [(2-\sqrt{3})u(x_1,\beta _1)+(\sqrt{3}-1)u(x_2,\beta _1)-u(x_3,\beta _1)-u(x_1,\beta _3)\\&\hspace{0.4cm}-(\sqrt{3}+1)u(x_2,\beta _3)+(2+\sqrt{3})u(x_3,\beta _3) +(\sqrt{3}-1)u(x_1,\beta _2)\\&\hspace{0.4cm}+2u(x_2,\beta _2)-(\sqrt{3}+1)u(x_3,\beta _2)\Big ], \end{aligned}$$

where $x_1\in \left( \frac{k}{m},\frac{k+\frac{1}{3}}{m}\right) ,$ $x_2\in \left( \frac{k+\frac{1}{3}}{m},\frac{k+\frac{2}{3}}{m}\right)$ and $x_3\in \left( \frac{k+\frac{2}{3}}{m}, \frac{k+1}{m} \right)$. Upon using Lipschitz condition on above expression, following inequality is obtained:

40 $$\begin{aligned} |a_{i,p}|&\le 3^{-j-2}\Big [|u(x_1,\beta _1)-u(x_1,\beta _2)|+|u(x_1,\beta _1)-u(x_1,\beta _3)|+\sqrt{3}|u(x_2,\beta _1)-u(x_1,\beta _1)|+|u(x_2,\beta _2)-u(x_1,\beta _2)|\nonumber \\&+|u(x_2,\beta _2)-u(x_3,\beta _2)|+\sqrt{3}|u(x_1,\beta _2)-u(x_3,\beta _2)|+(1+\sqrt{3})|u(x_3,\beta _3)-u(x_2,\beta _3)|+|u(x_3,\beta _3)-u(x_3,\beta _2)|\Big ],\end{aligned}$$

41 $$\begin{aligned} |a_{i,p}|&\le 3^{-j-2}\Big [ N_1|\beta _1-\beta _2|+N_2|\beta _1-\beta _3|+\sqrt{3}N_3|x_2-x_1|+N_4|x_2-x_1|+N_5|x_2-x_3|+\sqrt{3}N_6|x_1-x_3|\nonumber \\&+(1+\sqrt{3})N_7|x_3-x_2|+N_8|\beta _3-\beta _2|\Big ]. \end{aligned}$$

Setting $\hat{N}=max\left\{ N_1,N_2,\sqrt{3}N_3,N_4,N_5,\sqrt{3}N_6,(1+\sqrt{3})N_7,N_8 \right\}$, the following identity is derived:

42 $$\begin{aligned} |a_{i,p}|&\le 3^{-j-2}\frac{7\hat{N}}{3m}=3^{-j-2}\frac{N}{3m},\end{aligned}$$

43 $$\begin{aligned} |a_{i,p}|^2&\le \frac{N^2}{3^{4j+6}}. \end{aligned}$$

Upon substitution, (34) takes the form

44 $$\begin{aligned} \parallel E_{3M}\parallel _2^2&=K^2\sum _{i=3M+1}^{\infty }\sum _{p=3M+1}^{\infty }a_{i,p}^2 \end{aligned}$$

45 $$\begin{aligned}&\le K^2\sum _{i=3M+1}^{\infty }\sum _{p=3M+1}^{\infty }\frac{N^2}{3^{4j+6}}\end{aligned}$$

46 $$\begin{aligned}&=\frac{K^2N^2}{3^6}\sum _{j=J+1}^{\infty }\frac{4}{3^{4j}}3^{2j}\end{aligned}$$

47 $$\begin{aligned}&=\frac{K^2N^2}{2(3^{2J+6})}\end{aligned}$$

48 $$\begin{aligned} \parallel E_{3M}\parallel _2&=\frac{KN}{(9\sqrt{18})3^J}. \end{aligned}$$

It can be observed from (48) that, as $J\longrightarrow \infty$, $\parallel E_{3M}\parallel _2\longrightarrow 0$, which indicates the scale-3 HWM converges. *□*

## Conditioning analysis

A problem is termed ill-conditioned when small perturbations in the input data lead to disproportionately large variations in the output. The condition number quantifies how sensitive a solution is to these perturbations. Problems with small condition numbers are regarded as well-conditioned, whereas those with large condition numbers are considered ill-conditioned^36^. It is well known that the discrete Helmholtz problem becomes increasingly ill-conditioned as the wave number grows. Nevertheless, as noted in^37^, pp. 103], the condition number of the associated system matrix is generally smaller. In this work, we explicitly relate the ill-conditioning of the Helmholtz problem to the corresponding matrix condition number.

### Effective condition number for Haar wavelet method

Consider the linear algebraic system obtained by the method prescribed in Section Haar wavelet method for both Poisson and Helmholtz equations,

49 $$\begin{aligned} (\mathbf {R_x}+\mathbf {R_y})\textbf{u}=\mathbf {g(x_l,y_k)}-\mathbf {k_x}-\mathbf {k_y},\end{aligned}$$

50 $$\begin{aligned} (\mathbf {R_x}+\mathbf {R_y}+K^2\textbf{I})\textbf{u}=\mathbf {g(x_l,y_k)}-\mathbf {k_x}-\mathbf {k_y} \end{aligned}$$

respectively, where $\mathbf {R_x}+\mathbf {R_y}=\textbf{A}$ is a symmetric positive definite and $\mathbf {R_x}+\mathbf {R_y}+K^2\textbf{I}=\textbf{B}$ is symmetric indefinite matrices. $\textbf{u} \in \mathbb {R}^m$ and $\mathbf {g(x_l,y_k)}-\mathbf {k_x}-\mathbf {k_y}=\textbf{b} \in \mathbb {R}^m$ are unknown and known vectors. The absolute eigenvalues of $\textbf{A,B}$ can be ordered in descending sequence as $\lambda _1\ge \lambda _2\ge \cdots \ge \lambda _m$. The conventional condition number with respect to the 2-norm is defined as

51 $$\begin{aligned} Cond=\frac{\lambda _1}{\lambda _m}. \end{aligned}$$

In fact, when a perturbation $\delta \textbf{b}$ is introduced in $\textbf{b}$, the corresponding error $\delta \textbf{u}$ in the solution $\textbf{u}$ satisfies

52 $$\begin{aligned} \textbf{A}(\textbf{u}+\delta \textbf{u})=\textbf{b}+\delta \textbf{b}, \end{aligned}$$

where $\textbf{u}+\delta \textbf{u}=\tilde{\textbf{u}}$ is the approximate solution. In many practical applications, the right hand side vector $\textbf{b}$ is not arbitrary but restricted to a specific subspace. As a result, the relative errors arising from rounding are often significantly smaller than those predicted by the theoretical condition number *Cond* given in (51). A more realistic measure of this error is provided by the effective condition number, which was investigated by Christiansen and Hansen^38^.

The effective condition number plays a crucial role in the analysis of numerical methods such as the finite difference and finite element methods, particularly in situations involving local mesh refinement to resolve singularities. In such cases, the minimum mesh size (*Δ x*) may become extremely small, leading to a very large classical condition number, often of order $O(\Delta x^{-2})$, as noted in^39^. In contrast to this fact, the effective condition number yields a more accurate and typically much smaller estimate of the true numerical error. An analysis based on the effective condition number is also essential for wavelet-based methods. Although these methods often produce highly accurate solutions, they may exhibit severe numerical instability when assessed using the classical condition number, resulting in an apparent contradiction with the large value predicted by (51). The effective condition number resolves this discrepancy by providing a more meaningful measure of numerical stability.

#### Definition 1

Let $\textbf{A}$ be the symmetric matrix and $\lambda _1\ge \lambda _2\ge \cdots \ge \lambda _m$ be its eigenvalues. Then the effective condition number is given by

53 $$\begin{aligned} Cond_{Eff}=\frac{||\textbf{b}||}{\lambda _m\sqrt{\sum _{l=1}^{m}\frac{\gamma _l^2}{\lambda _l^2}}}, \end{aligned}$$

where $||\textbf{u}||=\sqrt{\sum _{l=1}^{m}\frac{\gamma _l^2}{\lambda _l^2}}$ and $\gamma _l=\textbf{w}_l^T\textbf{b}$ with $\textbf{w}_l$ is the corresponding eigenvector. The approximate effective condition number is given by

54 $$\begin{aligned} Cond_{\tilde{E}ff}=\frac{||\textbf{b}||}{\lambda _m ||\tilde{\textbf{u}}||}. \end{aligned}$$

Note that (53) needs all the eigenvectors $\textbf{w}_l$ to be calculated for $Cond_{Eff}$. By focusing exclusively on the eigenvector corresponding to the smallest eigenvalue, one may define a simplified form of the effective condition number. Due to the fact that,

55 $$\begin{aligned} ||\textbf{u}||=\sqrt{\sum _{l=1}^{m}\frac{\gamma _l^2}{\lambda _l^2}}=\sqrt{\sum _{l=1}^{m-1}\frac{\gamma _l^2}{\lambda _l^2}+\frac{\gamma _m^2}{\lambda _m^2}}\ge \sqrt{\frac{1}{\lambda _1^2}\sum _{l=1}^{m-1}\gamma _l^2+\frac{\gamma _m^2}{\lambda _m^2}}=\sqrt{\frac{||\textbf{b}||^2-\gamma _m^2}{\lambda _1^2}+\frac{\gamma _m^2}{\lambda _m^2}}, \end{aligned}$$

a simplified effective condition number from (53) can be defined as follows:

56 $$\begin{aligned} Cond_E=\frac{||\textbf{b}||}{\lambda _m\sqrt{\frac{||\textbf{b}||^2-\gamma _m^2}{\lambda _1^2}+\frac{\gamma _m^2}{\lambda _m^2}}}=\frac{||\textbf{b}||}{\sqrt{\left( ||\textbf{b}||^2-\gamma _m^2 \right) \left( \frac{\lambda _m^2}{\lambda _1^2}\right) +\gamma _m^2}}=\frac{||\textbf{b}||}{\sqrt{\left( \frac{\left( ||\textbf{b}||^2-\gamma _m^2 \right) }{Cond^2}\right) +\gamma _m^2}}, \end{aligned}$$

where *Cond* is as given in (51). It can be observed that $Cond_{Eff}\le Cond_E$. Specifically, provided that $\gamma _m \ne 0$, the simplest form of the effective condition number follows from (56) and is defined as

57 $$\begin{aligned} Cond_{EE}=\frac{||\textbf{b}||}{|\gamma _m|}, \end{aligned}$$

with $Cond_E\le Cond_{EE}$. If $\gamma _m$ is close to zero, the effective condition number $Cond_{EE}$ may become larger than *Cond*. Under these circumstances, the use of $Cond_{EE}$ is not meaningful and is therefore ignored. Furthermore, for $\gamma _m=0$, the simplified effective condition number $Cond_{E}$ coincides with the classical condition number.

### Preconditioner

**Fig. 2:**
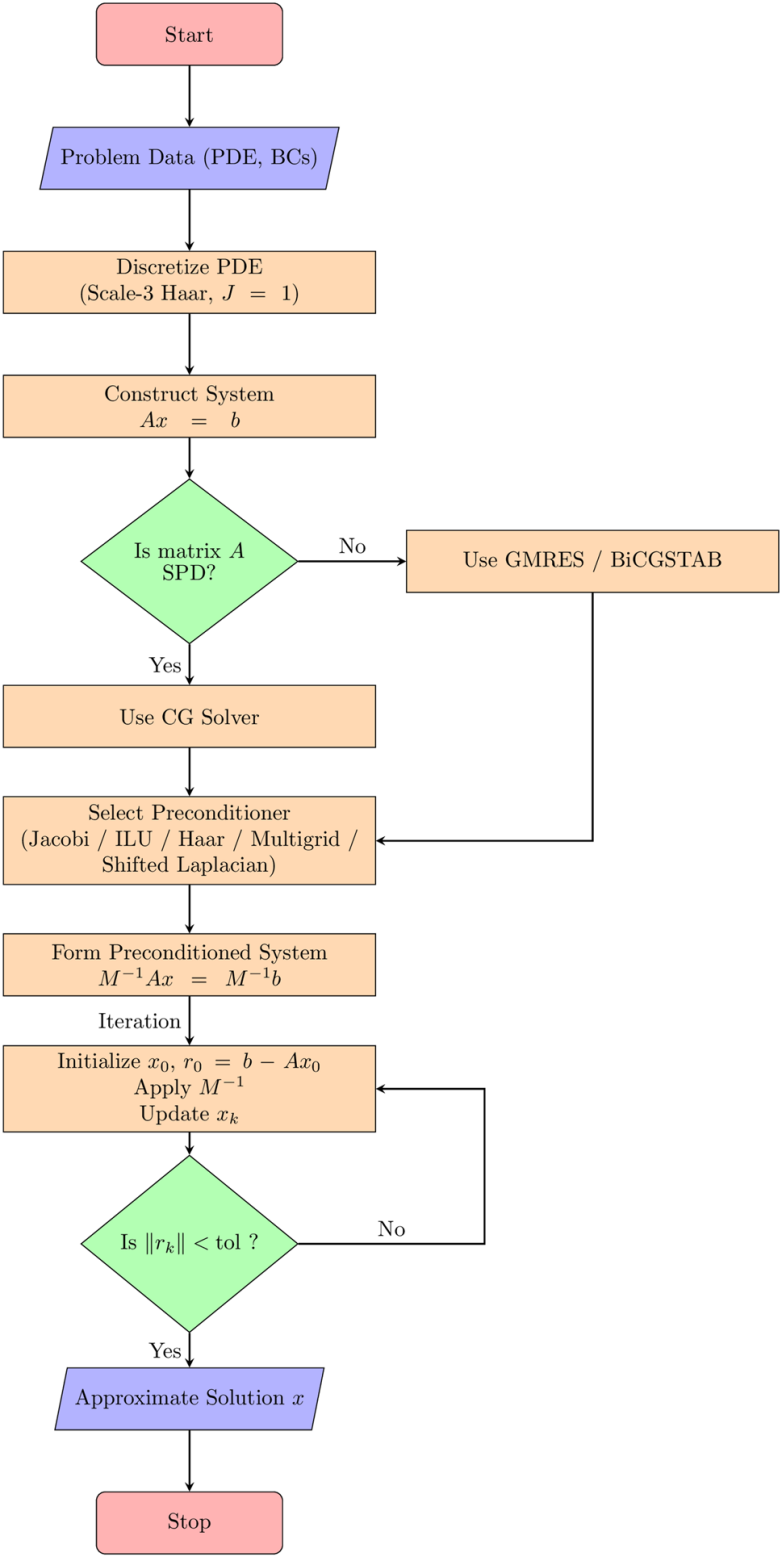
Pictorial illustration of the preconditioning framework and Krylov subspace solution procedure for the system *Ax=b* at given resolution level *J* (SPD - Symmetric Positive Definite and *k* - iteration index).

A preconditioner is a matrix *M* that approximates the inverse or key spectral properties of the system matrix *A* and is used to transform the linear system *Ax=b* into an equivalent system $M^{-1}Ax=M^{-1}b$ (commonly referred to as left preconditioner), with improved numerical properties. The primary goal of preconditioning is to reduce the condition number of the system and cluster the eigenvalues, thereby enhancing stability and accelerating convergence of iterative solvers. The discretizations of PDEs especially for large-scale, indefinite, or highly oscillatory problems (e.g., Helmholtz equations with large wave number) often lead to ill-conditioned linear systems. Such systems cause slow convergence or even stagnation of iterative methods. Preconditioning mitigates these issues by improving the spectral distribution of the coefficient matrix, making the system more amenable to efficient iterative solution. However, it is important to note that defining a universal preconditioner for a given problem is generally not feasible, as they are inherently problem specific and method dependent. As illustrated in Fig. 2, after discretizing the PDE and selecting an appropriate Krylov subspace solver based on whether the system matrix is SPD or not, the need for preconditioning is assessed. If the system exhibits instability or ill-conditioning, a suitable preconditioner is then identified, often through a guided trial and error approach, to achieve optimal performance.

Among wavelet-based preconditioners, incomplete discrete Haar wavelet transform are particularly attractive due to their low computational cost and less storage requirements, while still providing good convergence by efficiently capturing multiscale characteristics of the solution. Multigrid and algebraic multigrid preconditioners are popularly known as the most efficient techniques for large-scale linear systems, as they combine smoothing operations with coarse-grid correction to eliminate errors at different scales. These methods are typically implemented using *V*-cycle or *W*-cycle strategies, where relaxation schemes such as Jacobi or Gauss–Seidel are applied on each grid level, followed by restriction to coarser grids and prolongation back to finer grids. While geometric multigrid relies on explicit mesh hierarchies, AMG constructs the multilevel structure directly from the system matrix, making it well-suited for problems defined on unstructured grids. The shifted Laplacian preconditioner is especially effective for indefinite problems such as the Helmholtz equation. It is constructed by modifying the original operator through the addition of a complex or real shift, typically of the form $-\Delta -(K^2+i\alpha K^2)$ where the shift parameter *α* controls the amount of damping. This spectral shift improves the definiteness of the operator, thereby enhancing the convergence and robustness of Krylov subspace solvers.

### Krylov subspace methods

To elucidate the significance of preconditioning strategies, this section presents a concise overview of Krylov subspace methods. Although stationary iterative techniques have been employed since the era of Gauss, they have largely been superseded in modern numerical practice by Krylov subspace methods. These methods compute approximations to the solution vector *x* by projecting the problem onto a sequence of progressively enriched Krylov subspaces. The concept of Krylov subspaces is attributed to Aleksei Nikolaevich Krylov, who first introduced them in the context of analyzing oscillatory behavior in mechanical systems^40^. A Krylov subspace is defined as

$$\begin{aligned} K_m(A,r_0)=span\{r_0,Ar_0,A^2r_0,\ldots ,A^{m-1}r_0\}, \end{aligned}$$

where $A\in \mathbb {C}^{m\times m}$, $r_0\in \mathbb {C}^{m}$ and $r_0=b-Ax_0$ is the initial residual. For the linear system, begin with an initial guess $x_0$ and compute the initial residual $r_0=b-Ax_0$. Subsequent iterates $x_1,x_2,\ldots$ are then constructed such that

58 $$\begin{aligned} x_m-x_0\in K_m(A,r_0), \end{aligned}$$

thereby reducing the original problem to a projection onto a much lower dimensional subspace. This formulation is closely related to polynomial approximation theory. Specifically, the condition (58) holds if and only if the iterate can be expressed as $x_m=x_0+q_{m-1}(A)r_0$, where $q_{m-1}$ is a polynomial of degree at most *m-1*, that is, $q_{m-1} \in \mathscr {P}_{m-1}$, with $\mathscr {P}_m$ denoting the space of polynomials of degree no greater than *m*. As a direct consequence, the $m^{th}$ residual satisfies

$$\begin{aligned} r_m=b-Ax_m=(I-Aq_{m-1}(A))r_0=p_m(A)r_0,\hspace{0.3cm} p_m\in \mathscr {P}_m,\hspace{0.2cm} p(0)=1. \end{aligned}$$

At this stage, the manner in which the iterates are chosen within the Krylov subspaces has not been specified; it is precisely this selection criterion that differentiates the various Krylov subspace methods. Rather than discussing these selection strategies in detail, we instead focus on the available convergence theory and on the spectral properties of the preconditioned matrix associated with these methods particularly its eigenvalue distribution, and hence they are typically used in conjunction with suitable preconditioners to achieve fast and robust convergence. Consequently, it is natural to seek an equivalent transformed system that preserves the same solution while exhibiting more favorable spectral characteristics.

## Numerical experiments

The analysis is carried out using three model problems – one Poisson problem and two Helmholtz problems. The numerical algorithms were implemented in MATLAB R2025a and executed on a system equipped with an Apple Silicon M4 Pro chip and 24 GB of unified memory.

**Fig. 3:**
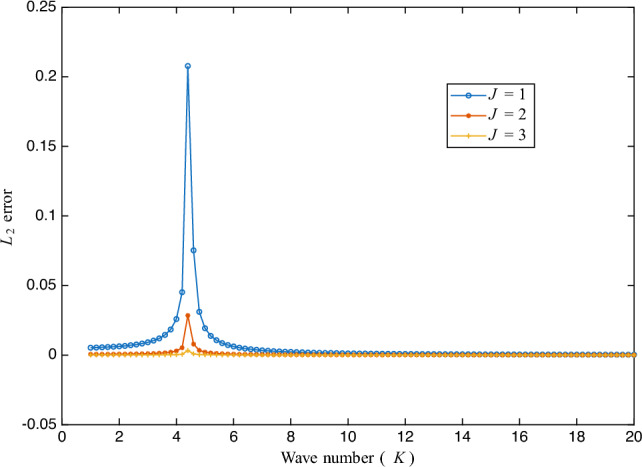
Variation of the $L_2$ error with respect to the wave number *K* for three different values of *J* in Problem 2.

**Fig. 4:**
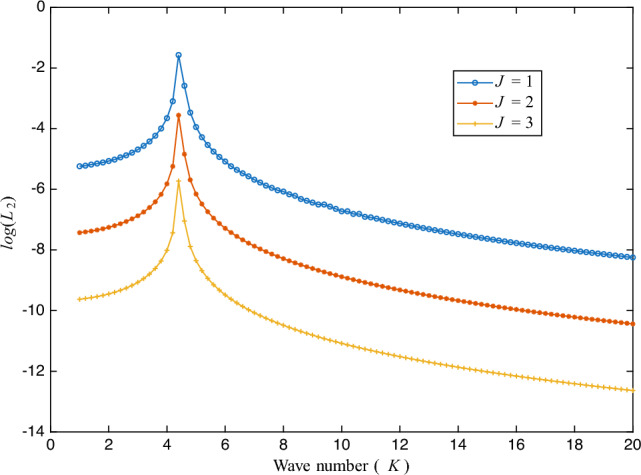
Dependence of the $log(L_2)$ error on the wave number *K* for three different values of *J* in Problem 2.

**Fig. 5:**
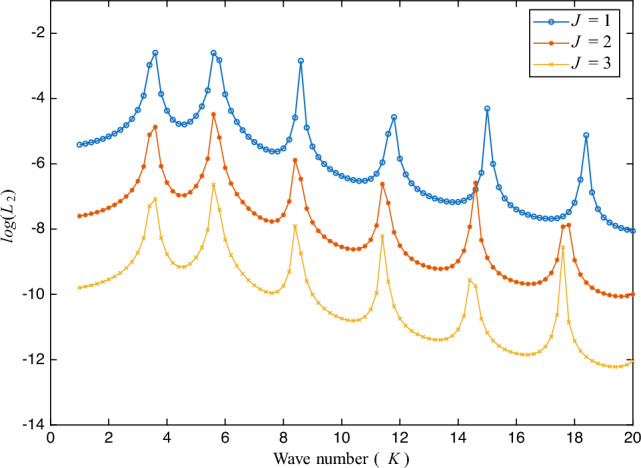
Dependence of the $log(L_2)$ error on the wave number *K* for three different values of *J* in Problem 3.

### In the absence of preconditioner

Problem 1. Consider a non homogeneous PDE as given bellow:

59 $$\begin{aligned} -\left( \frac{\partial ^2 u}{\partial x^2}+\frac{\partial ^2 u}{\partial y^2}\right) =\pi ^2x^\sigma (x-1)sin(\pi y)+[\sigma (\sigma -1)x^{\sigma -2}-\sigma (\sigma +1)x^{\sigma -1}]sin(\pi y) , \end{aligned}$$

**Table 1:** Analysis of condition and effective condition numbers for the smooth solution of (59) with *σ =2* on uniform difference grids.

Scale-2 Haar wavelet							
*J*	$\lambda _1$	$\lambda _m$	*Cond*	$Cond_{Eff}$	$Cond_{\tilde{E}ff}$	$Cond_E$	$Cond_{EE}$
1	256	20.226210	12.656854	1.386468	1.401930	1.501691	1.507646
2	1024	19.864870	51.548284	1.481545	1.422303	1.522032	1.522409
3	4096	19.770845	207.173737	1.425085	1.426138	1.525807	1.525830
4	16384	19.747131	829.690124	1.426749	1.427013	1.526668	1.526669
5	65536	19.741190	3319.759258	1.427160	1.427226	1.526878	1.526879
FDM^22^, pp. 225 Table 1]							
1	-	-	5.83	1.17	-	-	1.27
2	-	-	25.3	1.26	-	-	1.40
3	-	-	103	1.34	-	-	1.47
4	-	-	413	1.38	-	-	1.50

in the unit square domain with Dirichlet boundary condition *u=0*. For *σ =2*, using HW method the linear algebraic system as in (49) is obtained. For various values of *J*, the condition number, effective condition number, approximate effective condition number, and simplified effective condition number are reported in Tables 1 and 2 for both the scale-2 and scale-3 HW methods. The results indicate that, as *J* increases, the condition number grows significantly, leading to increased ill-conditioning of the system. Although a numerical solution is still obtained, the system becomes progressively less stable for larger values of *J*. The analytical solution for the above problem is given by $u(x,y)=x^{\sigma }(x-1)sin(\pi y)$.

**Table 2:** Analysis of condition and effective condition numbers for the smooth solution of (59) with *σ =2* on uniform difference grids using scale-3 HW.

*J*	$\lambda _1$	$\lambda _m$	*Cond*	$Cond_{Eff}$	$Cond_{\tilde{E}ff}$	$Cond_E$	$Cond_{EE}$
1	1296	19.838695	65.326874	1.420138	1.423430	1.523141	1.523377
2	11664	19.750334	590.572261	1.426526	1.426897	1.526554	1.526556
3	104976	19.740445	5317.813003	1.427211	1.427252	1.526904	1.526905

Problem 2. Consider the Helmholtz problem, as defined below:

60 $$\begin{aligned} \frac{\partial ^2 u}{\partial x^2}+\frac{\partial ^2 u}{\partial y^2}+K^2u=f(x,y); \hspace{1cm} (x,y)\in [0,1]\times [0,1] \end{aligned}$$

with *u(0,y)=u(1,y)=u(x,0)=u(x,1)=0* on the boundaries, where $f(x,y)=(K^2-2\pi ^2)sin(\pi x)sin(\pi y)$. The problem is solved using the above prescribed method, and the final system of equations are

61 $$\begin{aligned} (\mathbf {R_x}+\mathbf {R_y}+K^2\textbf{I})\textbf{u}=(K^2-2\pi ^2)\mathbf {sin(\pi x_l)sin(\pi y_k)}. \end{aligned}$$

Equation (61) is then solved using the Gauss-elimination method.

For the Helmholtz equation with wave numbers *K=4.44* and *K=20*, the condition number, effective condition number, approximate effective condition number, and simplified effective condition number corresponding to different values of *J* are presented in Tables 3 and 4 for both the scale-2 and scale-3 HW methods. The results demonstrate that increasing *J* leads to a substantial growth in the condition number, thereby exacerbating the ill-conditioning of the resulting linear system. While numerical solutions can still be obtained, the stability of the system deteriorates progressively for larger values of *J*. The analytical solution for this problem is given by $u_{ex}=sin(\pi x)sin(\pi y)$.

**Table 3:** Analysis of condition and effective condition numbers for smooth solutions to (60) under scale-2 HW discretization.

*J*	$\lambda _1$	$\lambda _m$	*Cond*	$Cond_{Eff}$	$Cond_{\tilde{E}ff}$	$Cond_E$	$Cond_{EE}$
*K=4.44*							
1	2.3628E+02	0.572594	4.6096E+02	0.149959	1	1	1
2	1.0042E+03	0.151270	6.6390E+03	0.169291	1	1	1
3	4.0762E+03	0.057245	7.1206E+04	0.447349	1	1	1
4	1.6364E+04	0.033531	4.8802E+05	0.763723	1	1	1
5	6.5516E+04	0.027590	2.3746E+06	0.928180	1	1	1
*K=20*							
1	3.8740E+02	153.38617	2.5256E+00	2.3405E+00	2.3379E+00	2.5256E+00	5.61E+15
2	8.8013E+02	6.805330	1.2933E+02	1.2934E+02	1.2933E+02	1.2933E+02	1.59E+15
3	3.1959E+03	10.320243	3.0968E+02	8.5294E+01	8.5291E+01	3.0968E+02	6.68E+16
4	1.5483E+04	3.580770	4.3242E+03	2.4582E+02	2.4582E+02	4.3242E+03	1.66E+15
5	6.4636E+04	5.230830	1.2356E+04	1.6828E+02	1.6828E+02	1.2356E+04	4.50E+14

**Table 4:** Analysis of condition and effective condition numbers for smooth solutions to (60) under scale-3 HW discretization.

*J*	$\lambda _1$	$\lambda _m$	*Cond*	$Cond_{Eff}$	$Cond_{\tilde{E}ff}$	$Cond_E$
*K=4.44*						
1	1.2762E+03	0.125095	1.0202E+04	0.204713	1	1
2	1.1644E+04	0.036734	3.1698E+05	0.697122	1	1
3	1.0495E+05	0.026845	3.9095E+06	0.953917	1	1
*K=20*						
1	8.8016E+02	1.073753	8.1970E+02	8.1979E+02	8.1970E+02	8.1970E+02
2	1.0764E+04	3.265396	3.2963E+03	2.6957E+02	2.6956E+02	3.2963E+03
3	1.0408E+05	7.656312	1.3593E+04	1.1497E+02	1.1497E+02	1.3593E+04

**Fig. 6:**
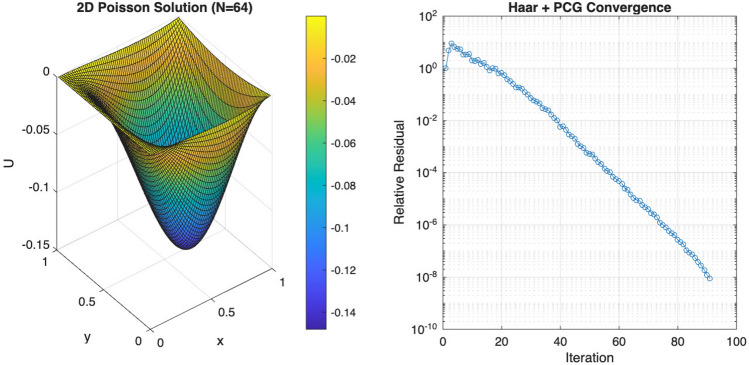
Numerical solution and convergence behavior of the Haar wavelet + PCG method for Problem 1 at resolution level *J=4* using scale-2 HW.

**Fig. 7:**
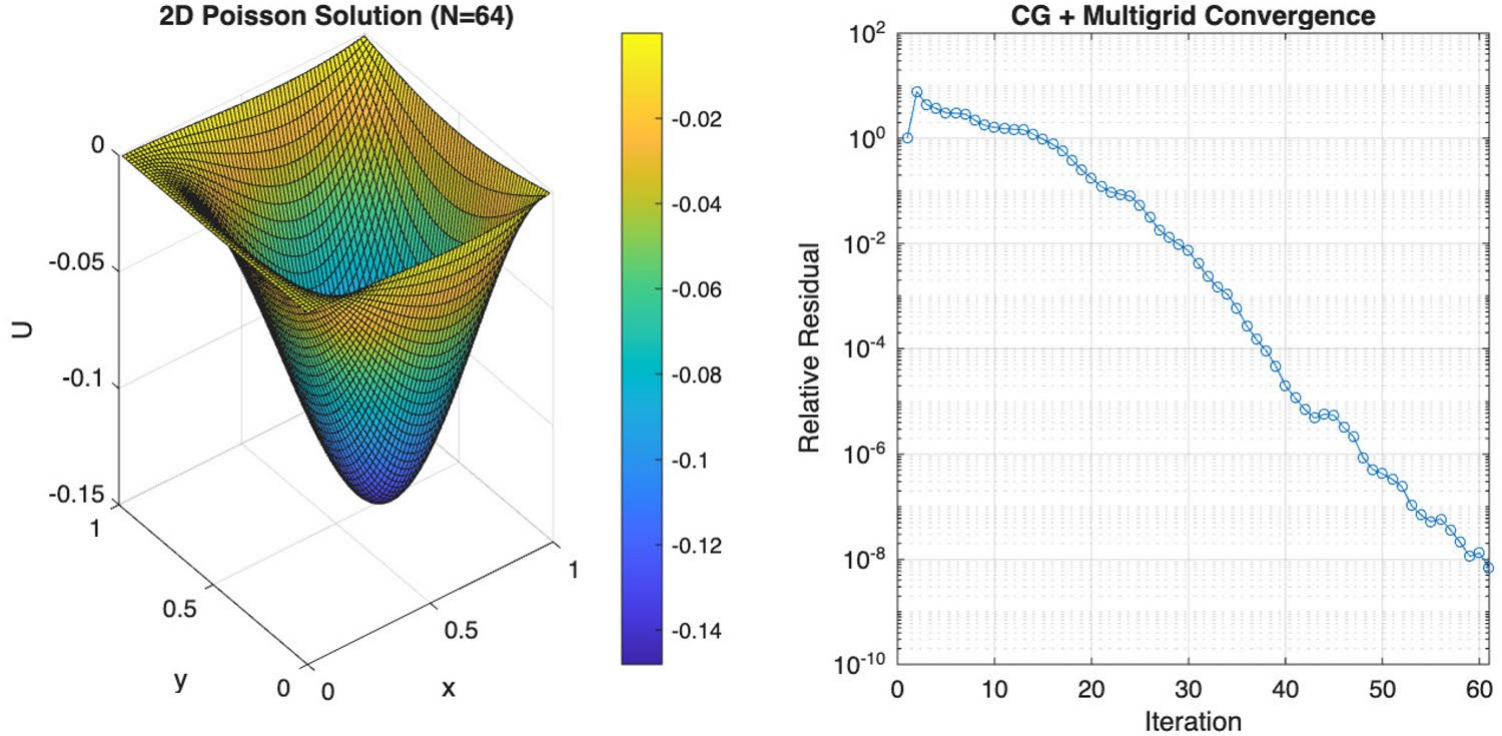
Numerical solution and convergence behavior of the Multigrid+CG method for Problem 1 for *J=4* with scale-2 HW.

**Fig. 8:**
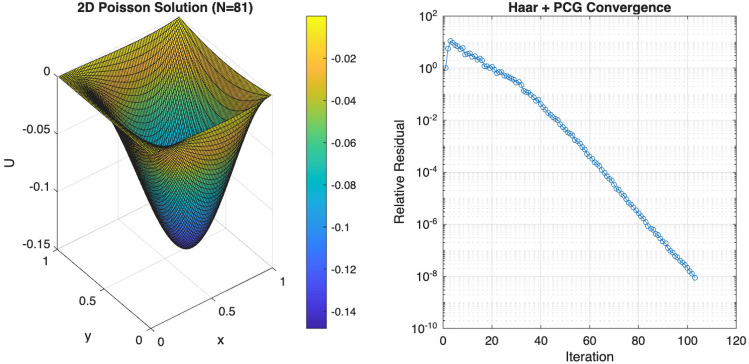
Numerical solution and convergence behavior of the Haar wavelet + PCG method for Problem 1 at resolution level *J=2* using scale-3 HW.

Problem 3. Let

62 $$\begin{aligned} \frac{\partial ^2 u}{\partial x^2}+\frac{\partial ^2 u}{\partial y^2}+K^2u=(K^2-2\pi ^2)cos(\pi x)sin(\pi y); \hspace{1cm} (x,y)\in [0,1]\times [0,1] \end{aligned}$$

defined on a unit square featuring a mixed set of boundary conditions: a Neumann condition on the left boundary, specified by $u_x(0,y)=0$, and Dirichlet conditions on the other three boundaries, given by *u(1,y)=-sin(π y)*, *u(x,0)=0* and *u(x,1)=0*. Utilizing the Kronecker tensor product in both the *x* and *y* spatial dimensions, the resulting extended approximation for the 2D domain takes the following form:

63 $$\begin{aligned} \begin{aligned} \mathbf {\frac{\partial ^2 u(x_l,y_k)}{\partial x^2}}&=\mathbf {(B_1\otimes I_y)u(x_l,y_k)} +\mathbf {(B_2\otimes I_y)f_1}\\&=\mathbf {R_x u(x_l,y_k)}+ \mathbf {k_x}, \end{aligned} \end{aligned}$$

64 $$\begin{aligned} \begin{aligned} \mathbf {\frac{\partial ^2 u(x_l,y_k)}{\partial y^2}}&=\mathbf {(I_x\otimes B_3)u(x_l,y_k)} +\mathbf {(I_x\otimes B_4)f_2}\\&=\mathbf {R_y u(x_l,y_k)}+ \mathbf {k_y}, \end{aligned} \end{aligned}$$

Here $\mathbf {f_1}$ and $\mathbf {f_2}$ are *6M× 1* column vectors where ${\mathbf {f_1}}^T=(0,0,\ldots ,0,-sin(\pi x_1),\ldots ,-sin(\pi x_{3M}))$ and $\mathbf {f_2}=0$. Addition of (63) and (64) leads to the following system of equations:

65 $$\begin{aligned} (\mathbf {R_x}+\mathbf {R_y}+K^2\textbf{I})\textbf{u}=(K^2-2\pi ^2)\mathbf {cos(\pi x_l)sin(\pi y_k)} -\mathbf {k_x}-\mathbf {k_y} . \end{aligned}$$

The aforementioned problem possesses an analytical solution given by $u_{ex}=cos(\pi x)sin(\pi y)$. The results presented in Table 5 indicate that the instability of the system becomes more severe as the resolution level increases, as evidenced by the drastic growth in the condition number.

**Table 5:** Analysis of condition and effective condition numbers for smooth solutions to (62) under scale-3 HW discretization.

*J*	$\lambda _1$	$\lambda _m$	*Cond*	$Cond_{Eff}$	$Cond_{\tilde{E}ff}$	$Cond_E$
*K=5.66*						
1	1.2542E+03	0.339766	3.6913E+03	3.6517E+02	4.3357E+02	6.5686E+02
2	1.1622E+04	0.074312	1.5639E+05	8.9440E+03	9.7676E+03	1.3702E+04
3	1.0493E+05	0.044363	2.3653E+06	7.8129E+04	7.9413E+04	1.0509E+05
*K=20*						
1	8.8622E+02	3.337911	2.6550E+02	1.0480E+02	1.0477E+02	2.5650E+02
2	1.1254E+04	7.206629	1.5616E+03	9.5515E+01	9.5512E+01	1.5616E+03
3	1.0045E+05	12.003405	8.7113E+03	2.8643E+02	2.8643E+02	8.7113E+03

The performance of the proposed scheme for different values of the wave number *K* was also investigated. Figures 3 and 4 demonstrate $L_2$ error and $log(L_2)$ aginst *K* while Fig. 5 represents the $log(L_2)$ aginst *K* with three different values of *J* for Problems 2 and 3, respectivley. As shown in Figures 3 and 4, for Problem 2 the method exhibits robust behavior with respect to variations in *K*, except in the range $3\le K\le 5$, where increased sensitivity is observed. In contrast, Fig. 5 indicates that the scheme applied to Problem 3 is more sensitive to changes in *K* when compared to Problem 2. However, for a fixed value of *J*, the overall error does not grow with increasing *K*. As observed in these figures, the method exhibits reduced accuracy at certain specific values of *K* for both problems. This observation can be understood through an eigenvalue analysis. The approximate eigenvalues for Problem 2, take the form $\lambda _{m,n}(2)=K^2-\pi ^2(m^2+n^2)$, whereas for Problem 3 the eigenvalues are approximated by $\lambda _{m,n}(3)=K^2-\pi ^2((m+\frac{1}{2})^2+n^2)$(see ref^41^.). For instance, near *K=4.44*, where $\lambda _{1,1}(2)\longrightarrow 0$, Problem 2 becomes unstable, while near *K=5.66*, where $\lambda _{1,1}(3)\longrightarrow 0$, Problem 3 exhibits instability, resulting in poor accuracy of the scheme. To overcome the instability arising from ill-conditioned systems and to improve the effectiveness of the method, an appropriate preconditioner was applied and solved the resulting system using a Krylov subspace method.

**Table 6:** Analysis of condition numbers using Haar and multigrid preconditioner for the smooth solution of (59) with *σ =2* on uniform difference grids using scale-2 HW.

*J*	*Cond* in the absence of preconditioner	*Cond* using scale-2 Haar preconditioner	No. of iterations	*Cond* using multigrid preconditioner	No. of iterations
1	12.656854	5.8478	7	1.0007	4
2	51.548284	19.8293	19	4.2596	18
3	207.173737	47.8348	35	6.7500	24
4	829.690124	109.9684	57	15.3509	37
5	3319.759258	244.5121	90	41.3597	60

**Table 7:** Analysis of condition numbers using Haar preconditioner for the smooth solution of (59) with *σ =2* on uniform difference grids using scale-3 HW.

*J*	*Cond* in the absence of preconditioner	*Cond* using scale-3 Haar preconditioner	No. of iterations
1	65.326874	29.7346	22
2	590.572261	107.5293	49
3	5317.813003	371.8161	102

**Table 8:** Analysis of condition numbers using shifted Laplacian preconditioner for the smooth solution of (60) on uniform difference grids using scale-2 and scale-3 HW.

*J*	*Cond* in the absence of preconditioner	*Cond* using preconditioner	No. of iterations	*Cond* in the absence of preconditioner	*Cond* using preconditioner	No. of iterations
*K=4.44*						
1	4.6096E+02	2.196886	1	1.0202E+04	1.374153	1
2	6.6390E+03	2.243297	1	3.1698E+05	1.390694	1
3	7.1206E+04	2.255086	1	3.9095E+06	1.425371	1
4	4.8802E+05	2.258032	1	-	-	-
5	2.3746E+06	2.267413	1	-	-	-
*K=20*						
1	2.5256E+00	16.4213	1	8.1970E+02	39.3297	1
2	1.2933E+02	39.2749	1	3.2963E+03	39.5136	1
3	3.0968E+02	39.4517	1	1.3593E+04	39.9375	1
4	4.3242E+03	39.5237	1	-	-	-
5	1.2356E+04	42.6873	1	-	-	-

**Fig. 9:**
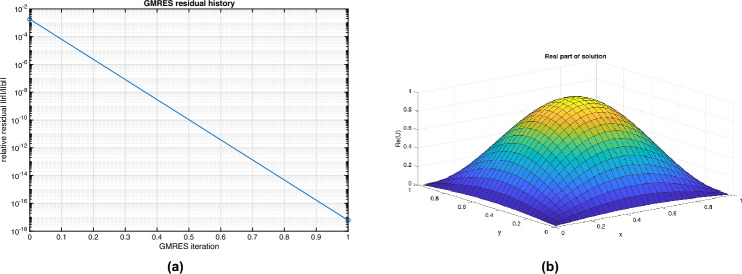
(**a**) GMRES residual plot of Problem 2 for *J=4* with scale-2 HW. and (**b**) GMRES solution of Problem 2 for *J=4* with scale-2 HW.

### In the presence of preconditoner

Among the various preconditioning strategies available in the literature, wavelet-based and multigrid-based preconditioners were used in conjunction with the Conjugate Gradient (CG) method for the Poisson problem. The corresponding results are reported in Table 6 for the scale-2 Haar wavelet discretization using both the scale-2 Haar preconditioner and the multigrid preconditioner, and in Table 7 for the scale-3 Haar wavelet discretization with the scale-3 Haar preconditioner. In the latter case, the multigrid preconditioner does not perform effectively when combined with the scale-3 Haar wavelet method. The results indicate that both preconditioners significantly improve the conditioning of the system and lead to good convergence of the CG method, with the multigrid preconditioner exhibiting superior performance compared to the Haar wavelet preconditioner.

In contrast, for the Helmholtz equation, a shifted Laplacian preconditioner combined with the GMRES method is adopted. The corresponding results are presented in Tables 8 and 9. Other combinations of preconditioners and Krylov subspace solvers were found to be less effective in achieving satisfactory convergence and reduced condition numbers. The shifted Laplacian GMRES approach yields a favorable condition number, resulting in a stable system that is close to being well-posed. The choice of these preconditioners is done by empirical performance, as there is no universally optimal preconditioner applicable to all classes of problems. However, the construction of a general preconditioner for a given method and problem is not possible.

**Fig. 10:**
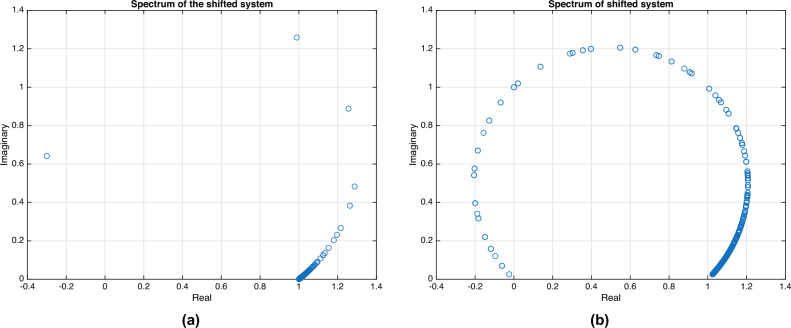
(**a**) Spectrum of preconditioned system of Problem 2 for *J=2*, *K=4.44* and (**b**) *K=20*, with scale-2 HW.

**Table 9:** Analysis of condition numbers using shifted Laplacian preconditioner for the smooth solution of (62) on uniform difference grids using scale-3 HW.

*J*	*Cond* in the absence of preconditioner	*Cond* using preconditioner	No. of iterations
*K=5.66*			
1	3.6913E+03	4.288440	1
2	1.5639E+05	4.305681	1
3	2.3653E+06	4.385936	2
*K=20*			
1	2.6550E+02	63.5743	1
2	1.5616E+03	63.8223	2
3	8.7113E+03	64.1535	2

**Fig. 11:**
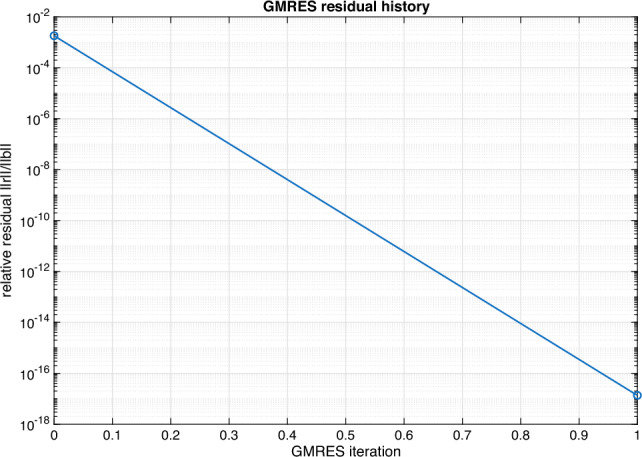
GMRES residual plot of Problem 2 for *J=2* with scale-3 HW.

**Fig. 12:**
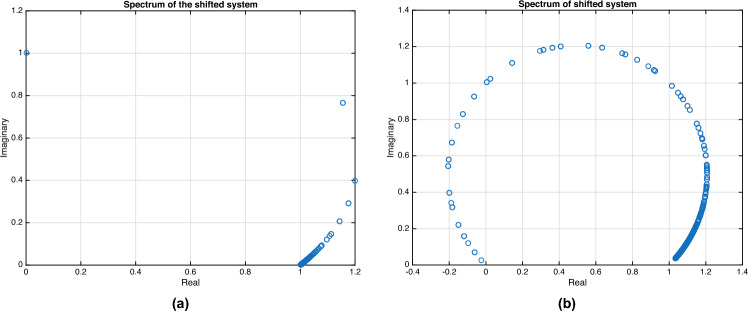
(**a**) Spectrum of preconditioned system of Problem 2 for *J=2*, *K=4.44* and (**b**) *K=20*, with scale-3 HW.

For the Poisson equation (59), Figures 6, 7, 8 illustrates the numerical solutions and the corresponding convergence behavior of the preconditioned Conjugate Gradient (CG) method. Figure 6 depicts the results for the scale-2 Haar wavelet discretization using the Haar wavelet preconditioned CG method. Figure 7 evaluates the same discretization but utilizes a multigrid preconditioned CG method. Whereas Fig. 8 extends the analysis to the scale-3 Haar wavelet discretization, showing the performance of the Haar wavelet preconditioned CG approach. As shown in the residual-versus-iteration plots across these figures, the residual decreases monotonically as iterations increase. This consistent trend confirms the numerical stability and proper convergence of the proposed preconditioning methods.

**Fig. 13:**
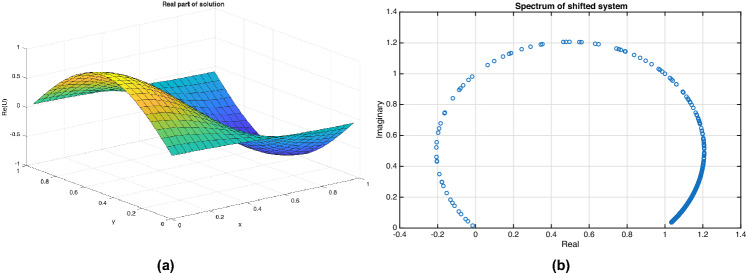
(**a**) GMRES solution plot of Problem 3 for *J=2* with scale-3 HW. and (**b**) Spectrum of preconditioned system of Problem 3 for *J=2* with scale-3 HW.

The GMRES residual history presented in Fig. 9a, together with the clustering of eigenvalues near unity in the spectrum of the shifted Laplacian preconditioned system shown in Fig. 10, demonstrates the effectiveness of the chosen preconditioner and solver for Problem 2 of the Helmholtz equation discretized using the scale-2 HW method. The corresponding numerical solution is illustrated in Fig. 9b. For the Helmholtz problem, the spectrum typically consists of the eigenvalues of the preconditioned operator $M^{-1}A$, where $A=\Delta +K^2I$ is the discrete Helmholtz operator and $M=\Delta +(1+i\alpha )K^2I$ denotes the shifted Laplacian preconditioner employed with GMRES. The tight clustering of eigenvalues near the point 1 in the complex plane indicates that the preconditioner provides a strong approximation to the original operator, such that the preconditioned system behaves as a small perturbation of the identity. The complex shift introduces artificial damping, which suppresses highly oscillatory and resonant modes inherent to the indefinite Helmholtz operator, thereby moving eigenvalues away from the origin and eliminating near-zero components that typically cause GMRES stagnation. From a numerical standpoint, this eigenvalue clustering implies that all error modes are preconditioned uniformly, enabling the GMRES residual polynomial to approximate a constant and resulting in rapid convergence. An improvement in both convergence behavior and spectral properties is observed when a scale-3 HW discretization is used in conjunction with the shifted Laplacian preconditioner and GMRES solver, as shown in Fig. 11 and Fig. 12 respectively. The GMRES solution for Problem 3 is presented in Fig. 13a, while the corresponding spectrum is shown in Fig. 13b. In this case, the majority of the eigenvalues are well separated from the origin and tightly clustered around unity, confirming enhanced numerical stability and convergence. The slight deviation of eigenvalues from unity can be attributed to discretization effects, boundary conditions, non-normality of the operator, and the finite choice of the shift parameter *α*, which balances damping strength against approximation accuracy.

### Computational complexity and scalability analysis

To provide further insight into the efficiency of the proposed method, a brief discussion on computational complexity and scalability is presented. For the scale-2 Haar wavelet method, at resolution level *J*, the number of unknowns scales as $N=O(2^J)$ in one dimension and $N=O(2^{2J})$ in two dimensions. The construction of the Haar operational matrices requires *O*(*N*) operations due to the simple piecewise-constant structure of the basis functions. Furthermore, the resulting system matrices are highly sparse, with the number of nonzero entries proportional to *O*(*N*). In the case of scale-3 Haar wavelets, although the number of basis functions remains proportional to $N=O(3^J)$, the support of each basis function slightly increases, leading to a marginal increase in the number of nonzero entries per row. As a result, the assembly cost and storage requirements remain *O*(*N*), but with a slightly larger constant factor compared to the standard Haar basis. This increase is compensated by improved approximation properties, allowing accurate solutions to be obtained at lower resolution levels.

Consequently, each iteration of an iterative solver such as GMRES requires *O*(*N*) operations, leading to an overall computational complexity of *O*(*kN*), where *k* denotes the number of iterations. In practice, the incorporation of an appropriate preconditioner significantly reduces *k*, often making it weakly dependent on *N*. This results in near-linear computational complexity with respect to the number of unknowns.

In comparison to classical finite difference or finite element methods, which may exhibit *O*(*NlogN*) or higher complexity depending on the solver strategy, the proposed Haar wavelet framework benefits from reduced computational cost due to sparsity and simple basis representation. Moreover, the scale-3 Haar approach can achieve comparable or higher accuracy at coarser resolutions, thereby effectively reducing the total number of unknowns required to achieve a given accuracy level. From a scalability perspective, the method demonstrates good performance as the resolution increases, since both storage and computational requirements grow approximately linearly with *N*. This makes the approach particularly suitable for large-scale simulations, where efficiency and memory usage are critical considerations.

## Conclusions

In this paper, we introduced a comprehensive condition number based analysis to assess and improve the numerical stability of elliptic partial differential equations, with particular emphasis on the Poisson equation and the highly ill-conditioned Helmholtz equation. The governing equations were discretized using scale-2 and scale-3 HW methods, and the resulting linear systems were analyzed using several conditioning measures, including the traditional condition number, the effective condition number, and the simplified effective condition number.

The numerical results clearly demonstrate that increasing the resolution level leads to a significant growth in the condition number, thereby deteriorating the stability of the system. In particular, small perturbations in the right hand side can induce large variations in the computed solution, especially for the Helmholtz problem. To address this issue, appropriate preconditioning strategies were employed, including Haar wavelet–based and multigrid preconditioners for the Poisson equation and a shifted Laplacian preconditioner for the Helmholtz equation. The preconditioned systems were solved using Krylov subspace methods, namely the Conjugate Gradient method for symmetric positive definite systems and the GMRES method for indefinite systems. The results confirm that the incorporation of suitable preconditioners significantly reduces the condition number, stabilizes the discrete systems, and leads to rapid and robust convergence of the Krylov solvers. In addition, a few importent observation from the present work are summarized below:

- It is not possible to generalize a matrix preconditioner for an entire class of problems. Preconditioners are largely problem specific, and their selection often requires trial and error.
- Spectral analysis of the preconditioned operators reveals favorable eigenvalue clustering, particularly for the shifted Laplacian preconditioned Helmholtz problem, which provides further insight into the observed convergence behavior and confirms the effectiveness of the proposed preconditioning strategies.
- Due to the inherent nonlinearity of the most practical problems, the discretized systems are often ill-conditioned or unstable. Consequently, even minimal errors in experimental data can propagate and lead to substantial deviations from the true solution, which necessitates the use of preconditioning.

Future research includes the extension of the present framework to nonlinear partial differential equations and time dependent problems, where conditioning issues are often more severe. The outcome of the research work aligns primarily with the United Nations Sustainable Development Goals SDG 9 (Industry, Innovation and Infrastructure) through the development of novel computational algorithms and numerical methodologies for solving PDEs arising in real-world applications along with the associated stability and conditioning analyses. Additionally, the study contributes to SDG 4 (Quality Education) by enriching the existing body of scientific knowledge in numerical analysis and scientific computing.

## Data Availability

All data generated or analysed during this study are included in this article.
